# Photodynamic therapy in glioblastoma: Detection of intraoperative inadvertent 5-ALA mediated photodynamic therapeutical effect after gross total resection

**DOI:** 10.3389/fonc.2022.1080685

**Published:** 2022-12-02

**Authors:** Abel Ferrés, Alberto Di Somma, Alejandra Mosteiro, Thomaz Eduardo Topczewski, Pedro Roldán, Leire Pedrosa, Diouldé Diao, Estela Pineda, Àngels Sierra, Joaquim Enseñat, José Juan González-Sánchez

**Affiliations:** ^1^ Department of Neurosurgery, Clínic Institute of Neurosciences, Hospital Clínic of Barcelona, Barcelona, Spain; ^2^ Faculty of Medicine and Health Sciences, University of Barcelona, Barcelona, Spain; ^3^ Department of Biomedicine, Faculty of Medicine, August Pi i Sunyer Biomedical Research Institute (IDIBAPS), Barcelona, Spain; ^4^ Servicio de Oncología Médica, Clinical Institute of Hematological and Oncological Diseases, Hospital Clinic of Barcelona, Barcelona, Spain; ^5^ Department of Medicine and Life Sciences, Facultad de Ciencias de la Salud y de la Vida, Universidad Pompeu Fabra, Barcelona, Spain

**Keywords:** photodynamic therapy, 5-aminolevulinic acid, glioblastoma, neurosurgical oncology, locorregional adjuvant therapy

## Abstract

**Introduction:**

Glioblastoma (GBM) remains the most frequent and lethal primary brain tumor in adults, despite advancements in surgical resection techniques and adjuvant chemo- and radiotherapy. The most frequent recurrence pattern (75-90%) occurs in the form of continuous growth from the border of the surgical cavity, thus emphasizing the need for locoregional tumor control. Fluorescence-guided surgical resection using 5-ALA has been widely implemented in surgical protocols for such tumors. Recent literature also highlights the applicability of 5-ALA-mediated photodynamic therapy to obtain locoregional tumor control further. This study aims to identify if 5-ALA mediated photodynamic therapeutic effect after gross total glioblastoma resection has inadvertently occurred due to the exposition of protoporphyrin IX charged peripheral tumoral cells to operative room light sources.

**Methods:**

Of 146 patients who were intervened from glioblastoma between 2015 and 2020, 33 were included in the present study. Strict gross total resection (without supralocal resection) had been accomplished, and adjuvant chemoradiotherapy protocol was administered. Two comparison groups were created regarding the location of the recurrence (group A: up to 1 centimeter from the surgical cavity, and group B: beyond 1 centimeter from the surgical cavity). The cutoff point was determined to be 1 centimeter because of the visible light penetrance to the normal brain tissue.

**Results:**

In univariate analysis, both groups only differed regarding 5-ALA administration, which was significantly related to a minor relative risk of presenting the recurrence within the first centimeter from the surgical cavity **(Relative Risk = 0,655 (95% CI 0,442-0,970), p-value=0,046)**. Results obtained in univariate analysis were corroborated posteriorly in multivariate analysis **(RR=0,730 (95% CI 0,340-0,980), p=0,017)**.

**Discussion:**

In the present study, a probable inadvertent 5-ALA photodynamic therapeutical effect has been detected in vivo. This finding widely opens the door for further research on this promising theragnostic tool.

## Introduction

Glioblastoma (GBM) (1) remains the most frequent (incidence of about 4-5 cases per 100000 inhabitants per year) and lethal (median overall survival time of 15 months) primary brain tumor in adults (2), despite the best surgical resection techniques and adjuvant chemo-radiotherapy (3, 4). Its highly infiltrating nature and tendency to recurrence primarily limits locoregional disease control and impedes cure, thus resulting in low survival rates (5, 6). Therefore, there is a need to identify and develop new treatments to increase locoregional disease control following surgical resection.

In this direction, locoregional photodynamic therapy has been progressively studied, especially *in vitro*. It has shown promising results in selective tumoral cell death, thus sparing normal brain parenchyma (7–14). Photodynamic therapy (PhT) is a two-step treatment that involves the administration of a photosensitizer agent (5-aminolevulinic acid; 5-ALA), which produces intracellular Protoporphyrin IX (PPIX) accumulation. PPIX is a fluorophore metabolite whose activation at a specific light wavelength (600-800 nm) generates oxidative stress and consequent cell death (10, 15–17).

It is well known that GBM cells can be found up to 4 centimeters beyond the border of radiologically or histologically identifiable tumor (18), and the most frequent recurrence pattern (75-90%) occurs in the form of continuous growth from the border of the surgical cavity (19–23). In association, visible white light (380-700 nm) penetrance in cerebral tissue may reach 1-centimeter depth (especially at 600 nm and favored in cases of low residual cell density in the surgical field (24)), and part of its spectrum is superposed for PPIX activation (25–28) resulting in its therapeutic effect.

Based on the previous statements, a minor recurrence rate within the first centimeter from the surgical cavity border may be hypothesized in patients affected by GBM after gross total resection (100% of the contrast-enhancing lesion) when 5-ALA has been administrated in conjunction with visible light exposure to the surgical field during the surgery.

The present study aims to determine if locoregional photodynamic therapy has been inadvertently produced *in vivo*, thus encouraging further investigation in humans.

## Materials and methods

### Study population

The central nervous system (CNS) tumor database of the Hospital Clínic de Barcelona, Spain, was queried to identify all patients treated for glioblastoma between 2015 and 2020. A total of 146 patients were initially identified. Patients finally included in the present study were those whose gross total resection of the contrast-enhancing lesion was achieved, resulting in 33 individuals. Supratotal resection (contrast-enhancing lesion plus 100% of hyperintense area in T2-weighted FLAIR) was not obtained. Variables included in the study were demographic characteristics, initial symptoms, initial functional status, tumor location, preoperative tumor volumetry, tumor superficiality classification (29), leptomeningeal dissemination, ependymal disease, 5-aminolevulinic acid administration (5-ALA), intraoperative adjuvants (neurophysiologic monitoring and intraoperative magnetic resonance imaging (MRI), surgical time, light exposure time, supratotal resection, adjuvant treatment (chemotherapy and radiotherapy protocols), location of the recurrence with respective to the surgical field (up to 1 cm vs. beyond 1 cm), and complications. Two comparison groups were created according to the location of the recurrence (Group A: up to 1 cm from the surgical field, and Group B: beyond 1 cm from the surgical field). Differences between groups have been initially evaluated through univariate analysis, focusing on differences regarding 5-ALA administration. After that, the differences encountered were corroborated using multivariate analysis. This study involving human participants was reviewed and approved by Barcelona’s Clínic hospital ethical board. According to legislation, participant informed consent was not necessary to be obtained because of the study’s retrospective nature, anonymized recorded clinical data, and the impossibility of identifying participants directly or through identifiers in study results.

### Location of the recurrence

The study’s principal objective was to evaluate if there are differences regarding the location of the recurrence concerning the surgical field in patients whose 5-ALA was administrated versus patients whose 5-ALA was not administrated and if an unnoticed photodynamic therapeutical effect could explain these differences after controlling for possible confounding variables.

**Figure 1:**
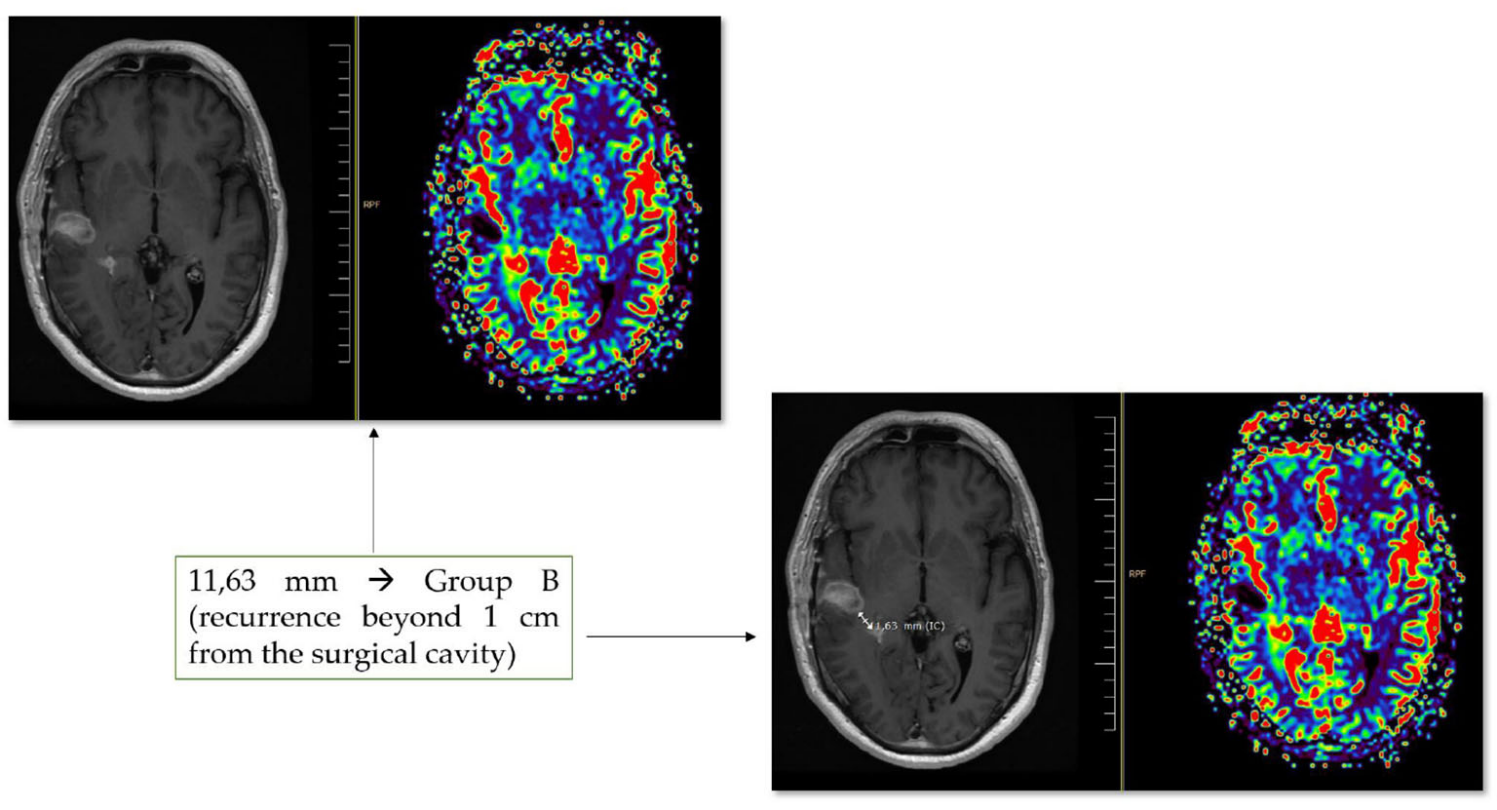
An example of the location of recurrence evaluation is provided. In this figure, a unique recurrence is detected at 11,63 mm from the surgical cavity. It allows us to classify the patient in group B MRI perfusion sequences may help both detect the recurrence and discard pathological gadolinium uptake at the margins of the surgical cavity.

The initial and residual tumors were measured onto volumetric MRI acquired through a 1,5 T scanner (Siemens) within the first 48 hours from the surgery, through a semiautomatic region of interest (ROI) analysis with Iplan cranial v.3.0 software (Brainlab^®^, Feldkirchen, Germany). T1-weighted gadolinium-enhanced and T2-weighted FLAIR sequences were used to define preoperative enhancing tumor volume and infiltrative volume outside the enhanced areas, respectively. Residual tumor volume was measured in postoperative MRI performed up to 48 hours from the surgery and co-registered with the preoperative dataset. Only patients presenting gross total resection of the contrast-enhancing lesion were included. A variable amount of infiltrating non-contrast enhancing tumor (hyperintense in T2-weighted FLAIR sequence) may have been resected. Still, in any of the included participants, FLAIR area resection reached 100%, thus not considered supratotal resection. For these patients, the follow-up MRI when recurrence after first-line treatment was detected was posteriorly evaluated. Radiologic tumor recurrence was defined according to the response assessment in neuro-oncology (RANO-HGG) 2010 criteria (30). The location of the recurrence from the surgical field was calculated using the nearest distance between the previous surgical field to the recurrent lesion (if solitaire) or the nearer recurrent lesion (if multiple) using the ruler tool of the radiological imaging software RAIM server DICOM viewer^®^ (UDIAT S.A., Sabadell, Barcelona). Patients were then categorized into the two groups previously described in the study population. An example is provided in **Figure 1**.

### Light exposure time

The retrospective nature of this study focused on evaluating a possible inadvertent 5-ALA photodynamic therapeutic effect in patients affected by GBM impeded a protocolized light delivery to the surgical field in terms of homogenous distribution, intensity, and time duration. Thus, the light was progressively delivered to the surgical cavity during tumor resection and hemostasis. In our study and being aware of this previously mentioned heterogenicity in light delivery, we considered light exposure time as the result of the duration between corticotomy and dural closure. This information was retrospectively obtained from surgical and anesthesia reports.

### Statistical analysis

Descriptive analysis was realized for both comparison groups. Continuous variables were described using median and range, and categoric variables were defined using absolute and relative frequencies. Comparative analysis was realized after that. For univariate analysis, differences between groups for continuous variables were evaluated using the Mann-Whitney U test and presented with the p-value. Differences between groups for categoric variables were assessed using the Chi-square test and given *via* relative risk (RR) and p-value. Multivariate analysis was calculated using logistic regression, and differences were presented with the p-value and RR.

## Results

Thirty-three patients from 146 initially identified to be treated for glioblastoma were finally included in the statistical analysis. Gross total resection (without supratotal resection) was achieved, and complete adjuvant therapy (chemo- and radiotherapy) was administered, thus homogenizing the sample.

The median age was 63,5 and 63 years for groups A and B, respectively, with male predominance in group A (11; 61,1%) and slight female predominance in group B (8; 53,3%). Initial symptoms were more frequent behavioral changes in group A and seizures in group B, without significant differences in initial functional status (median Karnofsky Performance Scale (KPS) of 95 and 90 for each group, respectively). Tumors were more frequently located in the temporal lobe (9; 50%) in group A and frontal (5; 33,3%) and parietal (4; 26,3%) lobes in group B, principally cortical positioned in both groups, without significant differences. Median preoperative volumetry was slightly different between groups: 25,43cc for group A and 30,64cc for group B, without reaching statistical significance. In most cases, neither leptomeningeal dissemination nor ependymal disease was detected. For group B, intraoperative MRI and neuromonitoring were more frequently needed as intraoperative adjuvants without reaching statistical significance. No significant differences were related to the tumor’s molecular characteristics (MGMT, IDH, ATRX, EGFR, TP53, and Ki67). All patients received adjuvant chemo- and radiotherapy without significant differences in the treatment protocol. Regarding chemotherapy, temozolomide was the first choice in both groups (94,4% and 100% for groups A and B, respectively). Regarding radiotherapy, hyperfractioned protocol was also the first option in both groups (90,4% and 86,7% for groups A and B, respectively). After treatment of the tumor, no significant differences were found between groups regarding hospital length of stay, complications, and functional status. Group’s characteristics are summarized in **Table 1**.

**Table 1:** Basal characteristics.

Variable			Group A (n=18)	Group B (n=15)	p-value
**Age**			63,5 (41-87)	63 (49-88)	0,2
**Sex**	Male		11 (61,1%)	7 (46,7%)	0,494
	Female		7 (38,9%)	8 (53,3%)	
**Initial symptoms**	Headache		1 (5,6%)	3 (20%)	0,153
	Seizure		2 (11,1%)	4 (26,7%)
	Hemiparesis/hemiplegia		2 (11,1%)	3 (20%)
	Behavioral changes		9 (50%)	3 (20%)
	Hemihypoesthesia/anesthesia		2 (11,1%)	0
	Aphasia		0	1 (6,7%)
	Facial paralysis		0	1 (6,7%)
	Mutism		2 (11,1%)	0
**Initial KPS**			95 (80-100)	90 (70-100)	0,539
**Tumor location**	Frontal		2 (11,1%)	5 (33,3%)	0,223
	Temporal		9 (50%)	2 (13,3%)
	Parietal		5 (27,8%)	4 (26,3%)
	Occipital		1 (5,6%)	2 (13,3%)
	Frontoparietal		0	1 (6,7%)
	Parietooccipital		1 (5,6%)	1 (6,7%)
**Superficiality**	Subventricular zone		0	0	0,641
	Cortex		14 (77,8%)	12 (80%)
	Subventricular zone and cortex		3 (16,7%)	3 (30%)
	None (white substance)		1 (5,6%)	0
**Preoperative tumor volumetry**			25,43cc (15,65-45,86)	30,64 (20,79-47,76)	0,776
**Leptomeningeal dissemination**	Yes		2 (11,1%)	2 (13,3%)	0,622
	No		15 (83,3%)	12 (80%)
**Ependymal disease**	Yes		0	1 (6,7%)	0,437
	No		18 (100%)	13 (86,7%)
**5-ALA (5-aminolevulinic acid)**	Yes		11 (61,1%)	14 (93,3%)	**0,046**
	No		7 (38,9%)	1 (6,7%)
**Light exposure time (min)**			135 (15-207)	84 (27-252)	0,166
**Intraoperative adjuvants**	iMRI		3 (16,7%)	8 (53,3%)	0,223
	Neuromonitoring		0	4 (26,7%)
	Awake		2 (11,1%)	0
**MGMT**	Methylated		10 (55,6%)	11 (73,3%)	0,704
	No methylated		6 (33,3%)	4 (26,7%)
**IDH**	Mutated		0	0	0,255
	Wild type		18 (100%)	15 (100%)
**ATRX**	ATRX +		0	1 (6,7%)	1
	ATRX -		4 (22,2%)	8 (53,3%)
**TP53**	Mutated		5 (27,8%)	8 (53,3%)	0,238
	No mutated		9 (50%)	4 (26,7%)
**EGFR**	Mutated		5 (27,8%)	10 (66,7%)	0,262
	Wild type		3 (16,7%)	1 (6,7%)
**Ki67**			30% (20-70%)	25% (5-40%)	0,112
**Chemotherapy**	Temozolomide		17 (94,4%)	15 (100%)	0,232
	Bevacizumab		1 (5,6%)	0
**Radiotherapy**	Hyperfractioned		17 (90,4%)	13 (86,7%)	0,193
	WBRT		1 (5,6%)	0
	Hypofractionated		0	2 (13,3%)
**Postoperative complications**	Immediate (first 24 hours)	Hematoma	1 (5,6%)	0	0,209
	Early (from first 24h up to 6th month)	Subacute hydrocephalus	0	1 (6,7%)	0,1
		Surgical wound infection	0	1 (6,7%)
		Hematoma	1 (5,6%)	0
	Late (beyond 6th month)	Hydrocephalus	1 (5,6%)	1 (6,7%)	0,282
**Hospital length of stay**			6 (4-21)	6,5 (3-25)	0,543
**Early KPS (up to 6th month)**			90 (50-100)	90 (70-100)	0,654
**Late KPS (after 6th month)**			80 (50-100)	70 (40-90)	0,235

Statistically significative values in bold.

When evaluating the relation between 5-ALA administration and the location of the recurrence, significant differences were found in the univariate analysis. 5-ALA administration was significantly related to a minor relative risk of presenting the recurrence within the first centimeter from the surgical cavity (0,655 95% CI 0,442-0,970; p=0,046) (**Table 2**). Afterward, the authors analyzed if light exposure time may be related to the location of the recurrence for the 5-ALA subgroup, and no significant differences were found (p=0,166) (**Table 3**).

**Table 2:** Univariate analysis.

Variable		5-ALA		RELATIVE RISK	p-Value
		Yes	No		
**Recurrence location**	Recurrence up to 1 cm from the surgical cavity	11 (61,1%)	7 (38,9%)	**0,655 (0,442-0,970)**	**0,046**
	Recurrence beyond 1 cm from surgical cavity	14 (93,3%)	1 (6,7%)	5,833 (0,805-42,253)	

Evaluation of the relationship between 5-ALA exposure and recurrence location.Statistically significative values in bold.

**Table 3:** Univariate analysis.

Variable		Light exposure time (median)	p-Value
**Recurrence location**	Up to 1 cm from the surgical cavity	136	0,166
	Beyond 1 cm from the surgical cavity	81

Evaluation of the relationship between light exposure time and recurrence location in the 5-ALA group.

The relation initially found between 5-ALA administration and minor relative risk to present the recurrence within the first centimeter from the surgical field was posteriorly evaluated in conjunction with other possible confounding variables in the multivariate analysis. Items included in the statistical analysis, different from 5-ALA administration, were superficiality classification, radiotherapy protocol administered, chemotherapy protocol administered, ependymal disease, leptomeningeal dissemination, and light exposure time. Again, only the relation between 5-ALA administration and negligible risk for the location of the recurrence up to the first centimeter from the surgical cavity reached statistical significance (0,730 95% CI 0,340-0,980); p=0,017) (**Table 4**).

**Table 4:** Multivariate analysis.

Variable	Recurrence location (up to 1 cm from surgical cavity vs. Beyond 1 cm)
**5-ALA**	**0,017 (RR=0,540; 95% CI 0,453-0,872) (a)**
**Superficiality classification**	0,299
**Radiotherapy protocol**	0,107
**Chemotherapy protocol**	0,283
**Ependymal disease**	0,169
**Leptomeningeal dissemination**	0,332

(a) relation between recurrence location beyond 1 cm from the surgical cavity and 5-ALA administration.Statistically significative values in bold.

## Discussion

Because the most frequent recurrence pattern in patients affected by HGG occurs in the form of continuous growth from the border of the surgical cavity, 5-ALA metabolites (protoporphyrin IX) can be activated at the visible light wavelength (380-700 nm), and its penetrance in cerebral tissue may reach up to 1 cm depth, the apparent 5-ALA photodynamic therapeutical effect may have been inadvertently produced on operated patients affected from glioblastoma when 5-ALA has been administered preoperatively, and tumoral intracellular protoporphyrin IX intraoperatively activated through operative room light sources. The present study aimed to analyze if this hypothesized 5-ALA photodynamic effect has been inadvertently produced in a cohort of patients affected by glioblastoma.

Considering the previously exposed statements, the authors hypothesized that the recurrence of patients with 5-ALA that was administrated preoperatively would be less probable located within the first centimeter from the surgical cavity. To accurately evaluate this hypothesis, confusion control is of utmost importance. Restrictive inclusion criteria, group comparativeness evaluation, and multivariate analysis were used. Our results have shown that recurrences were significantly less frequent within the first centimeter from the surgical cavity in the 5-ALA group, thus increasing the possibility of having detected an inadvertent *in vivo* 5-ALA photodynamic therapeutical effect in patients affected by GBM IDH wild-type. The presented results may have been facilitated, at least partially, due to the superficial location (cortical) of the tumors in most of the individuals included in the study. This location eases light exposure in the hole surgical cavity and promotes photodynamic reactions in patients in the 5-ALA group.

In addition to the principal analysis, differences regarding the location of the recurrence were evaluated concerning light exposure times only for the subgroup of patients whose 5-ALA was administered preoperatively. Paradoxically to what we would expect, the median light exposure time was minor in patients whose 5-ALA was administered and recurrence located beyond 1 cm from the surgical field, without reaching statistical significance. It is described in the literature that the dose of light delivered is essential to obtain maximum photobleaching of photosensitizer, thus resulting in optimal effectivity of the therapy. Advanced photobleaching is the fluence rate that causes more than 95% photobleaching of photosensitizer and is related to better results. For 5-ALA photodynamic therapy, advanced photobleaching is achieved at 4 mm from the surface of a light diffuser emitting power of 200 mW/cm for 1 hour (31–34). In the present study, median light exposure time surpasses 60 minutes in both groups; nevertheless, the power of the light source and surgical field exposure may not be constant and/or homogeneous between groups or individuals in the same group. Uncontrolled differences in reaching advanced photobleaching of the photosensitizer between groups may explain, at least partially, the paradoxical trend in results obtained.

In the literature, few studies have reported their preliminary experience with 5-ALA photodynamic therapy in patients affected by high-grade gliomas. Regarding its efficacy as a surgical adjuvant, one group combined fluorescence-guided surgery (FGS) and postoperative photodynamic therapy; 5-ALA photodynamic therapy was performed with an implanted catheter in patients with primary GBM on the day of FGS. Photodynamic therapy was then realized at 24-hour intervals for five sessions, and the results were compared with the control group (conventional surgical resection). Delayed mean tumor progression (8.6 vs. 4.8 months) and increased mean survival (52.8 vs. 24.6 weeks) were observed in the group that received both FGS and photodynamic therapy when compared with the control group (35). Another group combined 5-ALA FGS in patients affected by recurrent GBM and intracavitary 5-ALA photodynamic therapy through lase diffusers strategically positioned inside the resection cavity. They reported median progression-free survival of 6 months without an increase in surgical morbidity and postoperative complications (36). Regarding its efficacy as a sole rescue treatment for GBM recurrences, two groups report their experience. One of them evaluated the efficacy of this therapy in 10 patients affected by small (maximum diameter < 3 cm) circumscribed recurrent malignant gliomas. The 1-year survival rate was 60%, with a median survival of 15 months. The other group also evaluated the efficacy of this therapy in 15 patients affected by small newly diagnosed (maximum diameter < 4 cm). It was compared to GBM patients who underwent tumor resection alone. The interstitial photodynamic therapy group demonstrated a significantly longer median progression-free survival of 16 vs. 10.2 months and a 3-years survival of 56 vs. 21% (33, 37).

The two most extensive clinical studies have been recently published: On the one hand, Leroy et al., in 2021, described a series of 251 patients who underwent interstitial 5-ALA photodynamic therapy. Overall mortality was 1%, and transient and persistent morbidity was 5%. Tumor response after photodynamic therapy was 92%, progression-free survival was 14,5 months for *de novo* lesions and 14 months for recurrent ones, respectively, and overall survival was 19 months and eight months for the same groups, respectively (38). On the other hand, Lietke et al., in 2021, described 44 retrospectively evaluated patients after being also treated with interstitial photodynamic therapy. The median time to failure was 7.1 months, and the median progression-free survival was 13 months (39).

Although the publications are increasing on such attractive novel therapy, more studies are needed to further evaluate the efficacy, effectiveness, and safety of the 5-ALA photodynamic therapy for treating patients affected by GBM. The authors consider that the previously presented literature, in conjunction with the results mentioned in the present study, may justify efforts to investigate such a promising adjuvant and/or rescue therapy for the treatment of GBM. Our efforts have been primarily oriented toward evaluating its intraoperative efficacy because a lack of publications is evident in this specific treatment modality. In this direction, recently published preliminary results of the INDYGO trial postulated that 5-ALA photodynamic therapy delivered immediately after resection as adjuvant therapy for GBM is safe and may help to decrease the recurrence risk by targeting residual tumor cells in the resection cavity (40).

### Study limitations

Although necessary to provide the most reliable results, restrictive inclusion criteria limit its generalization. In addition, the study’s retrospective nature carries inherent bias despite efforts to control it as much as possible. Also, as mentioned previously in the methodology section, heterogenicity regarding light exposure time must be considered when analyzing related results. Nevertheless, despite this heterogenic light exposure, recurrence was less frequent within the first centimeter from the surgical cavity when 5-ALA was administered, and gross total resection was accomplished. Finally, although supramarginal resections have been excluded, smaller tumors located in non-eloquent areas may allow for a more aggressive resection, thus resulting in partial excision of the surrounding tissue. In the present study, achieving more significant resection of the peritumoral brain parenchyma may overestimate the photodynamic therapy’s effect due to the smaller amount of pathologic tissue (hyperintense in MRI FLAIR sequence) surrounding the surgical cavity.

## Conclusion

A possible inadvertent 5-ALA photodynamic therapeutical effect may have been detected in patients affected by GBM after gross total resection. These results, in conjunction with favorable data published in the literature and previously presented in the present study, encourage further investigation of this promising therapy as a surgical add-on after primary GBM resection or recurrence or as a sole rescue treatment in non-resectable or recurrent cases of this primary brain cancer.

## Data availability statement

The raw data supporting the conclusions of this article will be made available by the authors, without undue reservation.

## Ethics statement

This study involving human participants was reviewed and Photodynamic therapy in glioblastoma approved by Barcelona’s Clínic hospital ethical board. According to legislation, participant informed consent was not necessary to be obtained because of the study’s retrospective nature, anonymized recorded clinical data, and the impossibility of identifying participants directly or through identifiers in study results.

## Author contributions

AF and JG-S designed and led the present study. AF and AM developed the theory and performed the computations. AF and AM collected the clinical and economic data. AF, AM, AD, TT, PR, LP, DD, AS, and JG-S performed the analytical methods and interpreted them. AF drafted the manuscript. JG-S, AD, PR, AS, and JE critically revised the manuscript. JE gave institutional, material, and logistic support. All authors contributed to the article and approved the submitted version.

## Conflict of interest

The authors declare that the research was conducted in the absence of any commercial or financial relationships that could be construed as a potential conflict of interest.

## Publisher’s note

All claims expressed in this article are solely those of the authors and do not necessarily represent those of their affiliated organizations, or those of the publisher, the editors and the reviewers. Any product that may be evaluated in this article, or claim that may be made by its manufacturer, is not guaranteed or endorsed by the publisher.
